# *catena*-Poly[[(benzyl­diphenyl­phosphine-κ*P*)silver(I)]-μ-nitrato-κ^2^ *O*:*O*′-[(benzyl­diphenyl­phosphine-κ*P*)silver(I)]-μ-nitrato-κ^4^ *O*,*O*′:*O*′,*O*′′]

**DOI:** 10.1107/S2414314622007726

**Published:** 2022-08-12

**Authors:** Kariska Potgieter, Frederick P. Malan, Reinout Meijboom

**Affiliations:** a Department of Chemical Sciences, University of Johannesburg, PO Box 524, Auckland Park, 2006, Johannesburg, South Africa; b Department of Chemistry, University of Pretoria, Lynnwood Road, Hatfield, Pretoria, 0002, South Africa; Sunway University, Malaysia

**Keywords:** crystal structure, silver(I) complex, benzyl­diphenyl phosphine, nitrate

## Abstract

The structure of the title complex, [Ag_2_(NO_3_)_2_(C_19_H_17_P)_2_]_*n*_, reveals a chain emanating from the coordination of one phosphine ligand to each silver(I) cation, as well as the bis-monodentate coordination of a bridging nitrato ligand (per Ag atom) and the bis-bidentate coordination of another bridging nitrato ligand (per Ag atom). The distorted four-coordinate Ag atoms are characterized by bonding angles that notably deviate from the ideal tetra­hedral shape.

## Abstract 2

The title silver(I) benzyl­diphenyl­phosphine complex crystallizes as a one-dimensional chain propagating through nitrato anions bound to each adjacent silver cation *via* both bis-monodentate and bis-bidentate coordination.

## Graphical abstract

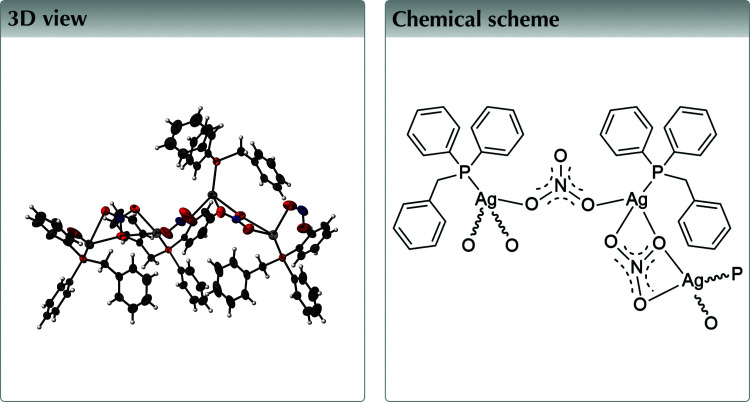

## Structure description

**Figure 1:**
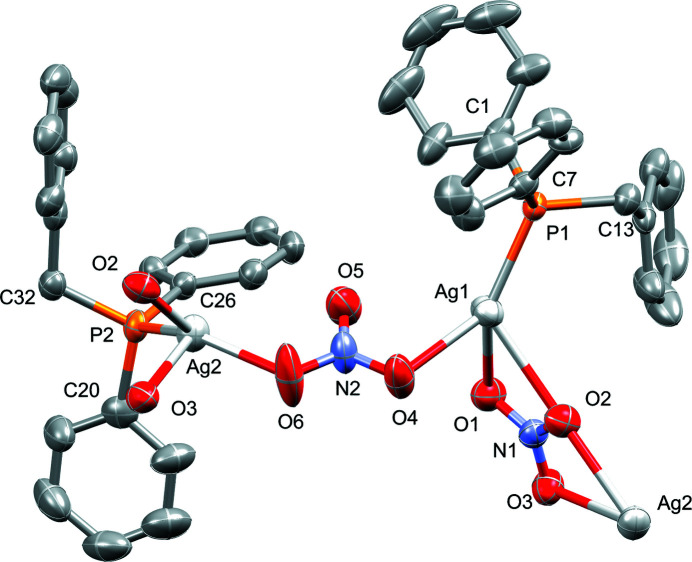
The mol­ecular structure of the asymmetric unit in the title compound showing displacement ellipsoids at the 50% probability level. Hydrogen atoms are omitted for clarity.

The solid-state mol­ecular structure of the title compound was established using single-crystal X-ray diffraction with data measured at 150 K. The complex crystallizes in the ortho­rhom­bic space group *Pna*2_1_ with *Z* = 4. The asymmetric unit contains two unique silver atoms, each with one benzyl­diphenyl phosphine ligand coordinated with bond lengths Ag1—P1 = 2.3506 (19) and Ag2—P2 = 2.3612 (19) Å. As seen in Fig. 1 ▸, each of the four-coordinate silver atoms are heavily distorted with bond angles P1—Ag1—O4 [129.6 (2)°], O1—Ag1—O4 [88.5 (3)°], P2—Ag1—O2 [121.08 (15)°], P2—Ag2—O2 [121.08 (15)°], O2—Ag2—O6 [96.0 (3)°] and P2—Ag2—O6 [142.8 (3)°]. Two unique nitrato groups bridge alternating silver atoms to form a polymeric chain. One nitrato group bridges Ag1 and Ag2 *via* three oxygen atoms (O1 and O2 bind to Ag1, O2 and O3 binds to Ag2) in a bis-bidentate fashion. This results in a near co-planar bond angle of Ag1—O2—Ag2 = 170.3 (5)°. The second nitrato group connects Ag1 to another Ag2 atom in a bis-monodentate fashion using only two oxygen atoms (O4 bonds to Ag1 and O6 bonds to Ag2). Differences in the respective Ag—O bond lengths of the two different nitrato groups are observed to fall within shorter [2.295 (7)–2.406 (7) Å] and longer [2.460 (6)–2.635 (7) Å] ranges.

**Figure 2:**
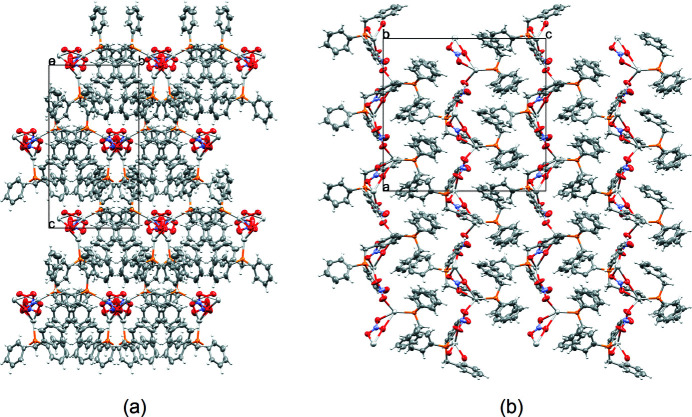
Perspective views along the (*a*) *a* and (*b*) *b* axes of the mol­ecular packing of the title compound.

The inorganic polymer packs in three dimensions as layers of one-dimensional ribbons when viewed along the *b* axis (Fig. 2 ▸); the chain has glide symmetry. Furthermore, the aromatic rings of the phosphine ligands then overlap in an adjacent layer to form a hydro­phobic layer in between Ag—NO_3_-containing layers.

## Synthesis and crystallization

Benzyl­diphenyl­phosphine (1 mmol) was dissolved in aceto­nitrile (10 ml). Silver nitrate (1 mmol) was dissolved in aceto­nitrile (10 ml). In order to obtain the given 1:1 molar ratio, the solutions were mixed. The resulting solution was heated to 353 K for approximately 2 h. The solution was removed from the heat and left to slowly cool. During the process of the slow evaporation of the solvent, clear colorless crystals started to form.

## Refinement

Experimental details including crystal data, data collection and structure refinement details are summarized in Table 1 ▸. The highest calculated residual electron density peak is 2.51 e^−^ Å^−3^ and is located 0.99 Å from Ag2, which is attributed to the presence of the strong absorber (Ag), as well as imperfections in the absorption correction process.

## Supplementary Material

Crystal structure: contains datablock(s) I. DOI: 10.1107/S2414314622007726/tk4081sup1.cif

Structure factors: contains datablock(s) I. DOI: 10.1107/S2414314622007726/tk4081Isup2.hkl

Supporting information file. DOI: 10.1107/S2414314622007726/tk4081Isup3.cdx

CCDC reference: 2193914

Additional supporting information:  crystallographic information; 3D view; checkCIF report

## Figures and Tables

**Table 1:** Experimental details

Crystal data	
Chemical formula	[Ag_2_(NO_3_)_2_(C_19_H_17_P)_2_]
*M*_r_	892.35
Crystal system, space group	Orthorhombic, *P* *n* *a*2_1_
Temperature (K)	150
*a*, *b*, *c* (Å)	18.0126 (3), 10.6251 (2), 19.2397 (3)
*V* (Å^3^)	3682.20 (11)
*Z*	4
Radiation type	Cu *K*α
μ (mm^−1^)	9.75
Crystal size (mm)	0.21 × 0.15 × 0.12

Data collection	
Diffractometer	XtaLAB Synergy R, DW system, HyPix
Absorption correction	Multi-scan (*CrysAlis PRO*; Rigaku OD, 2022 ▸)
*T*_min_, *T*_max_	0.665, 1.000
No. of measured, independent and observed [*I* > 2σ(*I*)] reflections	53360, 7741, 7352
*R*_int_	0.068
(sin θ/λ)_max_ (Å^−1^)	0.638

Refinement	
*R*[*F* ^2^ > 2σ(*F* ^2^)], *wR*(*F* ^2^), *S*	0.044, 0.120, 1.05
No. of reflections	7741
No. of parameters	451
No. of restraints	1
H-atom treatment	H-atom parameters constrained
Δρ_max_, Δρ_min_ (e Å^−3^)	2.51, −0.73
Absolute structure	Flack *x* determined using 3276 quotients [(*I* ^+^)−(*I* ^−^)]/[(*I* ^+^)+(*I* ^−^)] (Parsons *et al.*, 2013 ▸)
Absolute structure parameter	−0.009 (4)

Computer programs: *CrysAlis PRO* (Rigaku OD, 2022 ▸), *SHELXT* (Sheldrick, 2015*a* ▸), *SHELXL* (Sheldrick, 2015*b* ▸), and *OLEX2* (Dolomanov *et al.*, 2009 ▸).

## full crystallographic data

### Crystal data

**Table:**

[Ag_2_(NO_3_)_2_(C_19_H_17_P)_2_]	*D*_x_ = 1.610 Mg m^−^^3^
*M**_r_* = 892.35	Cu *K*α radiation, λ = 1.54184 Å
Orthorhombic, *P**n**a*2_1_	Cell parameters from 34740 reflections
*a* = 18.0126 (3) Å	θ = 4.6–78.2°
*b* = 10.6251 (2) Å	µ = 9.75 mm^−^^1^
*c* = 19.2397 (3) Å	*T* = 150 K
*V* = 3682.20 (11) Å^3^	Block, colourless
*Z* = 4	0.21 × 0.14 × 0.12 mm
*F*(000) = 1792	

### Data collection

**Table:**

XtaLAB Synergy R, DW system, HyPix diffractometer	7741 independent reflections
Radiation source: Rotating-anode X-ray tube, Rigaku (Cu) X-ray Source	7352 reflections with *I* > 2σ(*I*)
Mirror monochromator	*R*_int_ = 0.068
Detector resolution: 10.0000 pixels mm^-1^	θ_max_ = 79.5°, θ_min_ = 4.6°
ω scans	*h* = −18→22
Absorption correction: multi-scan (CrysAlisPro; Rigaku OD, 2022)	*k* = −13→13
*T*_min_ = 0.665, *T*_max_ = 1.000	*l* = −24→24
53360 measured reflections	

### Refinement

**Table:**

Refinement on *F*^2^	Hydrogen site location: inferred from neighbouring sites
Least-squares matrix: full	H-atom parameters constrained
*R*[*F*^2^ > 2σ(*F*^2^)] = 0.044	*w* = 1/[σ^2^(*F*_o_^2^) + (0.0747*P*)^2^ + 4.1236*P*] where *P* = (*F*_o_^2^ + 2*F*_c_^2^)/3
*wR*(*F*^2^) = 0.120	(Δ/σ)_max_ < 0.001
*S* = 1.05	Δρ_max_ = 2.51 e Å^−^^3^
7741 reflections	Δρ_min_ = −0.73 e Å^−^^3^
451 parameters	Absolute structure: Flack *x* determined using 3276 quotients [(*I*^+^)-(*I*^-^)]/[(*I*^+^)+(*I*^-^)] (Parsons *et al.*, 2013)
1 restraint	Absolute structure parameter: −0.009 (4)
Primary atom site location: dual	

### Special details

**Table:**

Geometry. All esds (except the esd in the dihedral angle between two l.s. planes) are estimated using the full covariance matrix. The cell esds are taken into account individually in the estimation of esds in distances, angles and torsion angles; correlations between esds in cell parameters are only used when they are defined by crystal symmetry. An approximate (isotropic) treatment of cell esds is used for estimating esds involving l.s. planes.
Refinement. All H atoms were placed in geometrically idealized positions and constrained to ride on their parent atoms.

### Fractional atomic coordinates and isotropic or equivalent isotropic displacement parameters (Å^2^)

**Table:**

	*x*	*y*	*z*	*U*_iso_*/*U*_eq_	
Ag1	0.20763 (3)	0.67285 (6)	0.57043 (3)	0.04561 (16)	
Ag2	0.49695 (3)	0.61627 (5)	0.44110 (5)	0.04420 (16)	
P1	0.20175 (9)	0.65229 (16)	0.69195 (10)	0.0293 (3)	
P2	0.52210 (9)	0.42137 (17)	0.38704 (10)	0.0337 (4)	
O1	0.1529 (4)	0.5950 (6)	0.4664 (3)	0.0513 (14)	
O3	0.0623 (3)	0.6720 (6)	0.4045 (3)	0.0487 (14)	
O2	0.0958 (4)	0.7638 (5)	0.4994 (3)	0.0461 (13)	
N1	0.1028 (4)	0.6746 (6)	0.4568 (3)	0.0374 (13)	
C26	0.4647 (3)	0.2889 (7)	0.4155 (4)	0.0321 (13)	
N2	0.3496 (4)	0.7223 (7)	0.4926 (4)	0.0487 (17)	
O5	0.3505 (5)	0.6175 (7)	0.5180 (4)	0.067 (2)	
O4	0.2935 (4)	0.7904 (7)	0.5001 (5)	0.068 (2)	
O6	0.4046 (4)	0.7612 (7)	0.4599 (6)	0.092 (3)	
C1	0.2459 (4)	0.5121 (6)	0.7253 (4)	0.0372 (15)	
C14	0.0614 (3)	0.5503 (8)	0.6926 (4)	0.0365 (15)	
C31	0.4020 (4)	0.3152 (7)	0.4557 (4)	0.0384 (15)	
H31	0.3917	0.3989	0.4701	0.046*	
C27	0.4797 (4)	0.1673 (7)	0.3947 (4)	0.0355 (14)	
H27	0.5217	0.1503	0.3665	0.043*	
C19	0.0502 (4)	0.4320 (8)	0.7222 (5)	0.0465 (19)	
H19	0.0705	0.4135	0.7666	0.056*	
C37	0.6669 (5)	0.2182 (9)	0.5650 (6)	0.0526 (19)	
H37	0.6626	0.1396	0.5883	0.063*	
C9	0.3472 (5)	0.9341 (9)	0.7401 (5)	0.054 (2)	
H9	0.3865	0.9766	0.7170	0.065*	
C11	0.2715 (5)	0.9051 (8)	0.8408 (5)	0.0471 (18)	
H11	0.2592	0.9286	0.8871	0.057*	
C28	0.4326 (4)	0.0685 (7)	0.4153 (4)	0.0395 (16)	
H28	0.4436	−0.0157	0.4023	0.047*	
C35	0.7050 (5)	0.4361 (9)	0.5645 (6)	0.052 (2)	
H35	0.7282	0.5052	0.5871	0.062*	
C13	0.1073 (4)	0.6497 (7)	0.7280 (4)	0.0360 (14)	
H13A	0.1096	0.6322	0.7785	0.043*	
H13B	0.0837	0.7330	0.7215	0.043*	
C33	0.6447 (4)	0.3482 (7)	0.4637 (4)	0.0355 (15)	
C10	0.3299 (6)	0.9636 (8)	0.8070 (6)	0.055 (2)	
H10	0.3583	1.0253	0.8308	0.066*	
C7	0.2484 (4)	0.7797 (7)	0.7387 (4)	0.0337 (14)	
C12	0.2310 (5)	0.8122 (8)	0.8074 (4)	0.0411 (16)	
H12	0.1917	0.7707	0.8309	0.049*	
C34	0.6760 (4)	0.4487 (8)	0.4988 (4)	0.0402 (16)	
H34	0.6774	0.5288	0.4768	0.048*	
C15	0.0299 (5)	0.5748 (11)	0.6288 (5)	0.054 (2)	
H15	0.0366	0.6554	0.6084	0.065*	
C38	0.6402 (4)	0.2314 (9)	0.4981 (5)	0.0477 (19)	
H38	0.6186	0.1612	0.4751	0.057*	
C36	0.6994 (5)	0.3173 (10)	0.5976 (5)	0.057 (2)	
H36	0.7186	0.3070	0.6432	0.069*	
C18	0.0101 (5)	0.3420 (10)	0.6875 (8)	0.062 (3)	
H18	0.0025	0.2619	0.7082	0.074*	
C30	0.3549 (4)	0.2173 (9)	0.4743 (5)	0.0460 (18)	
H30	0.3116	0.2347	0.5008	0.055*	
C6	0.2349 (5)	0.4681 (8)	0.7926 (5)	0.051 (2)	
H6	0.2010	0.5080	0.8233	0.061*	
C29	0.3703 (4)	0.0948 (8)	0.4548 (4)	0.0440 (18)	
H29	0.3380	0.0286	0.4685	0.053*	
C8	0.3063 (5)	0.8403 (8)	0.7055 (5)	0.0463 (19)	
H8	0.3186	0.8184	0.6590	0.056*	
C23	0.4602 (7)	0.4667 (12)	0.1562 (6)	0.072 (3)	
H23	0.4468	0.4801	0.1091	0.086*	
C20	0.5020 (4)	0.4340 (7)	0.2941 (4)	0.0406 (17)	
C2	0.2970 (5)	0.4527 (9)	0.6817 (6)	0.055 (2)	
H2	0.3046	0.4832	0.6358	0.066*	
C24	0.4162 (8)	0.5068 (11)	0.2077 (6)	0.074 (3)	
H24	0.3699	0.5445	0.1967	0.088*	
C22	0.5270 (7)	0.4042 (15)	0.1732 (6)	0.074 (4)	
H22	0.5583	0.3733	0.1374	0.089*	
C32	0.6180 (4)	0.3627 (7)	0.3912 (4)	0.0366 (14)	
H32A	0.6208	0.2803	0.3673	0.044*	
H32B	0.6510	0.4220	0.3662	0.044*	
C25	0.4366 (6)	0.4945 (10)	0.2755 (5)	0.061 (2)	
H25	0.4054	0.5280	0.3109	0.073*	
C4	0.3264 (8)	0.3052 (9)	0.7701 (9)	0.084 (4)	
H4	0.3540	0.2345	0.7857	0.101*	
C21	0.5468 (5)	0.3881 (11)	0.2420 (5)	0.056 (2)	
H21	0.5915	0.3452	0.2534	0.067*	
C17	−0.0197 (6)	0.3671 (14)	0.6222 (7)	0.075 (4)	
H17	−0.0458	0.3038	0.5973	0.090*	
C3	0.3366 (7)	0.3495 (11)	0.7051 (9)	0.081 (4)	
H3	0.3713	0.3096	0.6751	0.097*	
C16	−0.0106 (6)	0.4861 (17)	0.5941 (6)	0.079 (4)	
H16	−0.0326	0.5062	0.5506	0.095*	
C5	0.2766 (8)	0.3612 (10)	0.8135 (8)	0.075 (4)	
H5	0.2696	0.3279	0.8589	0.090*	

### Atomic displacement parameters (Å^2^)

**Table:**

	*U*^11^	*U*^22^	*U*^33^	*U*^12^	*U*^13^	*U*^23^
Ag1	0.0474 (3)	0.0544 (3)	0.0350 (2)	−0.0033 (2)	0.0018 (2)	0.0002 (3)
Ag2	0.0444 (3)	0.0335 (3)	0.0547 (3)	−0.0016 (2)	0.0037 (2)	−0.0057 (3)
P1	0.0242 (7)	0.0301 (8)	0.0335 (8)	0.0013 (6)	−0.0005 (6)	−0.0039 (7)
P2	0.0254 (7)	0.0310 (8)	0.0448 (9)	−0.0035 (7)	0.0029 (7)	−0.0018 (7)
O1	0.059 (4)	0.049 (3)	0.045 (3)	0.016 (3)	0.001 (3)	−0.004 (3)
O3	0.041 (3)	0.058 (4)	0.048 (3)	0.002 (3)	−0.002 (2)	−0.010 (3)
O2	0.061 (3)	0.034 (3)	0.044 (3)	0.005 (2)	0.002 (2)	−0.003 (2)
N1	0.037 (3)	0.034 (3)	0.041 (3)	0.004 (2)	0.002 (2)	0.006 (2)
C26	0.026 (3)	0.029 (3)	0.041 (4)	−0.002 (2)	−0.001 (3)	0.007 (3)
N2	0.038 (3)	0.038 (4)	0.069 (5)	−0.001 (3)	0.020 (3)	−0.004 (3)
O5	0.072 (5)	0.054 (4)	0.074 (5)	0.020 (3)	0.023 (4)	0.015 (3)
O4	0.060 (4)	0.050 (4)	0.092 (6)	0.016 (3)	0.035 (4)	0.017 (4)
O6	0.068 (5)	0.045 (4)	0.163 (10)	−0.003 (3)	0.071 (6)	0.003 (5)
C1	0.030 (3)	0.023 (3)	0.058 (4)	−0.001 (2)	−0.012 (3)	−0.008 (3)
C14	0.022 (3)	0.052 (4)	0.036 (3)	−0.004 (3)	0.004 (3)	−0.008 (3)
C31	0.032 (3)	0.038 (4)	0.045 (4)	0.001 (3)	−0.001 (3)	0.002 (3)
C27	0.030 (3)	0.037 (4)	0.039 (4)	−0.006 (3)	0.000 (3)	0.006 (3)
C19	0.031 (3)	0.045 (4)	0.063 (5)	−0.002 (3)	−0.004 (3)	−0.009 (4)
C37	0.044 (4)	0.058 (5)	0.055 (5)	0.010 (4)	0.005 (4)	0.011 (5)
C9	0.054 (5)	0.042 (4)	0.067 (6)	−0.020 (4)	−0.004 (4)	−0.003 (4)
C11	0.060 (5)	0.037 (4)	0.044 (4)	0.000 (4)	−0.011 (4)	−0.003 (3)
C28	0.039 (4)	0.034 (4)	0.046 (4)	−0.011 (3)	−0.010 (3)	0.005 (3)
C35	0.043 (4)	0.053 (5)	0.059 (5)	0.010 (4)	0.001 (4)	−0.019 (5)
C13	0.030 (3)	0.035 (4)	0.042 (4)	0.001 (3)	−0.001 (3)	−0.004 (3)
C33	0.023 (3)	0.037 (3)	0.047 (4)	−0.002 (3)	0.003 (3)	−0.002 (3)
C10	0.062 (5)	0.036 (4)	0.068 (6)	−0.011 (4)	−0.020 (5)	0.000 (4)
C7	0.034 (3)	0.026 (3)	0.041 (4)	0.002 (2)	−0.006 (3)	0.001 (3)
C12	0.046 (4)	0.043 (4)	0.035 (4)	−0.001 (3)	−0.009 (3)	−0.004 (3)
C34	0.031 (3)	0.038 (4)	0.052 (4)	0.005 (3)	−0.003 (3)	−0.002 (3)
C15	0.036 (4)	0.082 (6)	0.043 (4)	−0.018 (4)	0.002 (3)	−0.004 (5)
C38	0.036 (4)	0.044 (4)	0.063 (5)	−0.005 (3)	0.003 (3)	0.009 (4)
C36	0.051 (5)	0.077 (7)	0.044 (4)	0.028 (5)	0.003 (4)	0.002 (4)
C18	0.037 (4)	0.048 (5)	0.102 (9)	−0.008 (4)	0.000 (5)	−0.021 (6)
C30	0.027 (3)	0.059 (5)	0.052 (4)	−0.001 (3)	0.006 (3)	0.014 (4)
C6	0.055 (5)	0.032 (4)	0.066 (5)	−0.013 (3)	−0.026 (4)	0.007 (4)
C29	0.037 (4)	0.045 (4)	0.050 (5)	−0.011 (3)	−0.010 (3)	0.015 (3)
C8	0.045 (4)	0.037 (4)	0.057 (5)	−0.009 (3)	0.003 (4)	0.001 (4)
C23	0.078 (7)	0.079 (8)	0.059 (6)	−0.022 (6)	−0.022 (5)	0.029 (6)
C20	0.046 (4)	0.028 (4)	0.048 (4)	−0.019 (3)	−0.005 (3)	0.006 (3)
C2	0.038 (4)	0.048 (5)	0.079 (7)	0.011 (3)	−0.015 (4)	−0.021 (5)
C24	0.095 (8)	0.063 (6)	0.064 (7)	0.010 (6)	−0.020 (6)	0.016 (6)
C22	0.060 (6)	0.113 (10)	0.050 (6)	−0.028 (6)	0.014 (5)	−0.004 (6)
C32	0.030 (3)	0.035 (3)	0.045 (4)	−0.004 (3)	0.003 (3)	0.005 (3)
C25	0.077 (6)	0.050 (5)	0.054 (5)	0.014 (5)	−0.021 (5)	0.002 (4)
C4	0.088 (8)	0.030 (4)	0.135 (12)	0.019 (5)	−0.056 (8)	−0.012 (6)
C21	0.036 (4)	0.084 (7)	0.049 (5)	−0.008 (4)	0.001 (4)	0.003 (4)
C17	0.046 (5)	0.111 (10)	0.070 (7)	−0.025 (6)	0.002 (5)	−0.047 (7)
C3	0.069 (7)	0.050 (6)	0.125 (12)	0.029 (5)	−0.033 (7)	−0.022 (7)
C16	0.052 (5)	0.138 (13)	0.048 (5)	−0.043 (7)	−0.001 (4)	−0.021 (7)
C5	0.092 (8)	0.044 (5)	0.090 (9)	−0.019 (5)	−0.047 (7)	0.022 (6)

### Geometric parameters (Å, º)

**Table:**

Ag1—P1	2.3506 (19)	C37—C38	1.381 (14)
Ag1—O1	2.380 (6)	C37—C36	1.358 (15)
Ag1—O4	2.406 (7)	C9—C10	1.360 (15)
Ag2—P2	2.3612 (19)	C9—C8	1.407 (12)
Ag2—O2^i^	2.460 (6)	C11—C10	1.384 (14)
Ag2—O6	2.295 (7)	C11—C12	1.386 (12)
P1—C1	1.806 (7)	C28—C29	1.384 (12)
P1—C13	1.838 (8)	C35—C34	1.375 (14)
P1—C7	1.830 (7)	C35—C36	1.417 (15)
P2—C26	1.830 (7)	C33—C34	1.383 (11)
P2—C20	1.829 (9)	C33—C38	1.409 (11)
P2—C32	1.838 (7)	C33—C32	1.484 (11)
O1—N1	1.251 (9)	C7—C12	1.400 (11)
O3—N1	1.243 (9)	C7—C8	1.382 (11)
O2—Ag2^ii^	2.460 (6)	C15—C16	1.366 (15)
O2—N1	1.260 (8)	C18—C17	1.39 (2)
C26—C31	1.398 (10)	C30—C29	1.382 (13)
C26—C27	1.379 (11)	C6—C5	1.420 (14)
N2—O5	1.216 (10)	C23—C24	1.338 (19)
N2—O4	1.249 (10)	C23—C22	1.412 (19)
N2—O6	1.245 (10)	C20—C25	1.389 (13)
C1—C6	1.391 (13)	C20—C21	1.376 (13)
C1—C2	1.396 (12)	C2—C3	1.383 (15)
C14—C19	1.394 (12)	C24—C25	1.362 (15)
C14—C13	1.504 (10)	C22—C21	1.381 (15)
C14—C15	1.377 (12)	C4—C3	1.35 (2)
C31—C30	1.389 (11)	C4—C5	1.36 (2)
C27—C28	1.406 (10)	C17—C16	1.38 (2)
C19—C18	1.372 (13)		
			
P1—Ag1—O1	141.80 (17)	C18—C19—C14	120.4 (10)
P1—Ag1—O4	129.6 (2)	C36—C37—C38	120.1 (9)
O1—Ag1—O4	88.5 (3)	C10—C9—C8	119.4 (9)
P2—Ag2—O2^i^	121.08 (15)	C10—C11—C12	120.0 (9)
O6—Ag2—P2	142.8 (3)	C29—C28—C27	119.6 (8)
O6—Ag2—O2^i^	96.0 (3)	C34—C35—C36	118.2 (9)
C1—P1—Ag1	114.2 (3)	C14—C13—P1	110.4 (5)
C1—P1—C13	105.1 (4)	C34—C33—C38	118.4 (8)
C1—P1—C7	103.5 (3)	C34—C33—C32	120.6 (7)
C13—P1—Ag1	114.8 (3)	C38—C33—C32	121.0 (7)
C7—P1—Ag1	113.5 (2)	C9—C10—C11	121.1 (8)
C7—P1—C13	104.5 (3)	C12—C7—P1	122.9 (6)
C26—P2—Ag2	115.7 (2)	C8—C7—P1	117.6 (6)
C26—P2—C32	104.9 (3)	C8—C7—C12	119.3 (7)
C20—P2—Ag2	109.2 (3)	C11—C12—C7	119.7 (8)
C20—P2—C26	103.7 (3)	C35—C34—C33	121.8 (8)
C20—P2—C32	104.7 (4)	C16—C15—C14	121.7 (11)
C32—P2—Ag2	117.3 (3)	C37—C38—C33	120.5 (8)
N1—O1—Ag1	100.8 (5)	C37—C36—C35	120.9 (10)
N1—O2—Ag2^ii^	99.5 (4)	C19—C18—C17	120.6 (12)
O1—N1—O2	119.0 (7)	C29—C30—C31	120.9 (7)
O3—N1—O1	121.9 (7)	C1—C6—C5	117.2 (11)
O3—N1—O2	119.0 (6)	C30—C29—C28	120.1 (7)
C31—C26—P2	117.9 (5)	C7—C8—C9	120.4 (9)
C27—C26—P2	121.6 (5)	C24—C23—C22	118.9 (10)
C27—C26—C31	120.4 (6)	C25—C20—P2	117.0 (7)
O5—N2—O4	119.6 (7)	C21—C20—P2	124.8 (7)
O5—N2—O6	119.7 (7)	C21—C20—C25	118.3 (9)
O6—N2—O4	120.6 (8)	C3—C2—C1	120.2 (12)
N2—O4—Ag1	106.5 (5)	C23—C24—C25	121.3 (11)
N2—O6—Ag2	115.7 (6)	C21—C22—C23	120.0 (11)
C6—C1—P1	123.0 (6)	C33—C32—P2	112.4 (5)
C6—C1—C2	120.1 (8)	C24—C25—C20	121.3 (11)
C2—C1—P1	116.7 (7)	C3—C4—C5	120.4 (10)
C19—C14—C13	121.8 (7)	C20—C21—C22	120.2 (10)
C15—C14—C19	118.3 (8)	C16—C17—C18	118.8 (9)
C15—C14—C13	119.8 (8)	C4—C3—C2	120.5 (13)
C30—C31—C26	119.1 (7)	C15—C16—C17	120.1 (11)
C26—C27—C28	120.0 (7)	C4—C5—C6	121.6 (13)

Symmetry codes: (i) *x*+1/2, −*y*+3/2, *z*; (ii) *x*−1/2, −*y*+3/2, *z*.
